# Threat-sensitive anti-predator defence in precocial wader, the northern lapwing *Vanellus vanellus*

**DOI:** 10.1007/s10211-016-0236-1

**Published:** 2016-05-19

**Authors:** Natalia Królikowska, Jakub Szymkowiak, Rebecca Anne Laidlaw, Lechosław Kuczyński

**Affiliations:** 1 Department of Avian Biology and Ecology, Faculty of Biology, Adam Mickiewicz University, Umultowska 89, 61-614 Poznań, Poland; 2 School of Biological Sciences, University of East Anglia, Norwich Research Park, Norwich, NR4 7TJ UK; 3 South Iceland Research Centre, University of Iceland, Bankavegi, IS-800 Selfoss, Iceland

**Keywords:** Harm to offspring hypothesis, Parental investment theory, Predator recognition, Reproductive value of offspring hypothesis

## Abstract

Birds exhibit various forms of anti-predator behaviours to avoid reproductive failure, with mobbing—observation, approach and usually harassment of a predator—being one of the most commonly observed. Here, we investigate patterns of temporal variation in the mobbing response exhibited by a precocial species, the northern lapwing (*Vanellus vanellus*). We test whether brood age and self-reliance, or the perceived risk posed by various predators, affect mobbing response of lapwings. We quantified aggressive interactions between lapwings and their natural avian predators and used generalized additive models to test how timing and predator species identity are related to the mobbing response of lapwings. Lapwings diversified mobbing response within the breeding season and depending on predator species. Raven *Corvus corax*, hooded crow *Corvus cornix* and harriers evoked the strongest response, while common buzzard *Buteo buteo*, white stork *Ciconia ciconia*, black-headed gull *Chroicocephalus ridibundus* and rook *Corvus frugilegus* were less frequently attacked. Lapwings increased their mobbing response against raven, common buzzard, white stork and rook throughout the breeding season, while defence against hooded crow, harriers and black-headed gull did not exhibit clear temporal patterns. Mobbing behaviour of lapwings apparently constitutes a flexible anti-predator strategy. The anti-predator response depends on predator species, which may suggest that lapwings distinguish between predator types and match mobbing response to the perceived hazard at different stages of the breeding cycle. We conclude that a single species may exhibit various patterns of temporal variation in anti-predator defence, which may correspond with various hypotheses derived from parental investment theory.

**Electronic supplementary material:**

The online version of this article (doi:10.1007/s10211-016-0236-1) contains supplementary material, which is available to authorized users.

## Introduction

Predation constitutes the most important cause of reproductive failure in the majority of bird species (Ricklefs 1969; Montgomerie and Weatherhead 1988; Lima and Dill 1990; Martin 1995). Birds engage in various forms of anti-predator behaviour (Caro 2005) to enhance the chance of successful breeding. The most frequent are nest concealment and cryptic behaviours (Lima and Dill 1990), reducing the number of eggs under increased risk of nest predation (Eggers et al. 2006), nesting near protector species (Lima and Dill 1990), feigning injury behaviours (Caro 2005), changing their risk sensitivity depending on the risk and the option for re-nesting (Schneider and Griesser 2015) and active nest defence, like mobbing (Caro 2005; Lima and Dill 1990). Mobbing—observation, approach and usually harassment of a predator by prey birds with alarm calls and direct physical attacks (Caro 2005)—may effectively deter predators from nesting areas, thus ensuring higher offspring survival (Knight and Temple 1986; Caro 2005; Kontiainen et al. 2009). However, mobbing also involves some costs, as it potentially increases predation risk to defending parents or even attracts other predators into an area due to its conspicuousness (Krama and Krams 2005; Krams et al. 2007). Furthermore, mobbing is costly in terms of time and energy expenditure, as it reduces the time available for other activities (Lima and Dill 1990; Caro 2005). Consequently, parents should trade off the investment in current reproduction (anti-predator behaviour) and the ability to invest in the future (Clutton-Brock 1991; Stearns 1992).

The relationship between investment in current and future reproduction constitutes a life-history trade-off (Coleman and Gross 1991; Stearns 1992; Ghalambor and Martin 2001). Parental investment theory has yielded three hypotheses to explain patterns of temporal variation in eggs or chicks defence during the breeding season. The ‘reproductive value of offspring’ hypothesis assumes that the intensity of anti-predator defence matches the reproductive value of offspring. Thus, parents should take greater risks and increase mobbing intensity with increasing clutch and chick age (Andersson et al. 1980; Montgomerie and Weatherhead 1988). According to the predictions of the ‘harm to offspring’ hypothesis, defence intensity should peak when offspring are more vulnerable to the temporary suspension of parental care (Dale et al. 1996; Listøen et al. 2000; Fernández and Llambías 2013). The third hypothesis takes into account that for birds nesting in a multi-predator environment, different predators constitute different threats (Montgomerie and Weatherhead 1988; Lima and Dill 1990; Palestis 2005; Lima 2009). Some predators threaten the broods only temporarily (i.e., they feed mainly either on eggs or chicks), while others may potentially depredate them at all stages of the breeding cycle (e.g. Teunissen et al. 2008). The ‘threat-sensitive predator avoidance’ hypothesis suggests that individuals distinguish between different types of risk (some predators are hazardous only for the offspring while other pose risk only for adults or for both) and adjust anti-predator response to the perceived hazard posed by a given predator (Helfman 1989; Schneider and Griesser 2013). Such a flexible anti-predator behaviour allows individuals to reduce the costs associated with brood defence and is exhibited by several bird species (e.g. Montgomerie and Weatherhead 1988; Mathot et al. 2009; Strnad et al. 2012).

The majority of avian studies on offspring defence have focused on altricial (Krama et al. 2012; reviewed in Montgomerie and Weatherhead 1988; Caro 2005) or semi-precocial species (Clode et al. 2000; Whittam and Leonard 2000; Palestis 2005). While it has been suggested that the temporal patterns of the intensity of brood defence and willingness of birds to mob the predator may differ for precocial species (Barash 1975; Brunton 1990), empirical studies are relatively scarce (Brunton 1990; Larsen et al. 1996; Sordahl 2004; Mathot et al. 2009). Precocial species allocate their parental investments differently and face different trade-offs than altricial ones due to differences in the parental care system. Such differences may shape patterns of anti-predator response. Moreover, the hypotheses derived from the parental investment theory were mostly considered separately (Brown et al. 2006; Rhoades and Blumstein 2007; Mathot et al. 2009; Crawford et al. 2012), and these studies explored patterns of temporal variation in defence concerning a narrow range of predator taxa. Evaluating brood defence behaviour throughout the breeding season in precocial species, having mobile and dispersed chicks, is critical to gaining a wider understanding of anti-predator behaviour.

The majority of waders (including the northern lapwing *Vanellus vanellus*) consistently lay four eggs (Maclean 1972; Walters 1984). Thus, in this group, there is no relationship between defence intensity and clutch size or clutch volume (Rytkönen et al. 1995; Ruusila and Pöysa 1998; Kis et al. 2000), contrary to altricial species, in which anti-predator response often constitutes a function of brood size (Jónsson and Gunnarsson 2010) or parents adjust the number of eggs according to the risk of predation (Eggers et al. 2006; Zanette et al. 2011). In waders, the reproductive value of offspring increases with its age, but the offspring vulnerability likely peaks around hatching (Galbraith 1988; Montgomerie and Weatherhead 1988; Brunton 1990; Johansson and Blomqvist 1996; Weggler 2009). Chicks of waders (solely precocial species) are relatively self-reliant and demand markedly less parental attention than altricial young (Walters 1982, 1984; Schekkerman et al. 2009). The self-feeding activity of chicks may expose them to predation (Schekkerman et al. 2009), and parents likely defend them more vigorously than eggs, also due to the nests of many species being relatively well concealed (Šálek and Cepáková 2006). Older offspring that thermoregulate independently are more mobile and able to disperse (Weggler 2009), and thus have better survival and reproductive prospects than younger ones.

The northern lapwing (hereafter referred to as lapwing) is a ground-nesting wader that primarily inhabits extensively managed farmland dominated by pastures and marshes or wet meadows with short vegetation. Populations of lapwing have been declining in most European countries in recent decades (Krebs et al. 1999; Donald et al. 2001; Newton 2004). These declines have resulted primarily from low reproductive performance due to the interactive effects of habitat alterations and predation (Vickery et al. 2001; Evans 2004; Wilson et al. 2004; MacDonald and Bolton 2008; Teunissen et al. 2008). Red fox *Vulpes vulpes* and corvids likely cause the majority of breeding failures and represent the main predators of lapwing (Parr 1993; MacDonald and Bolton 2008; Teunissen et al. 2008; Laidlaw et al. 2015). It is well-known that corvids (e.g. Baines 1990; Berg 1992; Luginbuhl et al. 2001; Olsen 2003), in particular crows (e.g. Šálek and Šmilauer 2002; Bolton et al. 2007; Schekkerman et al. 2009) and ravens *Corvus corax* (e.g. Ewins et al. 1986; Marquiss and Booth 1986; Byrkjedal 1987), are the main, highly skilled avian predators of both eggs and chicks of waders. Gulls, storks and harriers may prey on birds’ eggs and chicks (Fletcher et al. 2005; Amar et al. 2008; Teunissen et al. 2008; Schekkerman et al. 2009, pers. observation) but in most regions represent rather incidental predators of waders clutches.

Where conditions allow, lapwings nest in aggregations—semi-colonies, where breeding is largely synchronized between pairs, and neighbouring birds collectively defend broods against predators by mobbing (Cramp and Simmons 1983; Elliot 1985; Kis et al. 2000). Although several studies have documented the aggressive anti-predator behaviour of lapwings and its effectiveness (e.g. Elliot 1985; Berg et al. 1992), little is known about its temporal variation during the breeding season (Kis et al. 2000).

We investigated temporal variation in the mobbing response during brood defence in lapwing against several avian predator species, based on observations of natural behaviour. Specifically, we investigated whether lapwings (i) increase the mobbing response against predators with offspring age (the reproductive value of offspring hypothesis), (ii) increase the mobbing response around and just after hatching (the harm to offspring hypothesis) and (iii) flexibly adjust mobbing response in relation to predator species (the threat-sensitive predator avoidance hypothesis).

## Methods

### Study site

Field studies were carried out in 1993, in central Poland, in the Warta River Valley (52° 10′ N, 17° 54′ E). The study site was a 2 km^2^, compact and approximately round in shape, regularly flooded nitrophilous grassland used as a pasture for cattle and domestic geese. The margins of the study plot were surrounded by tree lines, which forced lapwings to breed on a relatively small area in the centre of the plot, where nests were evenly distributed.

Based on our own observations and literature data (Kis et al. 2000; Schekkerman et al. 2009), we predict that the following avian predators recorded in the study area could be considered as potentially dangerous for lapwing offspring (eggs or chicks): white stork *Ciconia ciconia*, marsh harrier *Circus aeruginosus*, hen harrier *Circus cyaneus*, Montagu’s harrier *Circus pygargus*, common buzzard *Buteo buteo*, rough-legged buzzard *Buteo lagopus*, Eurasian sparrowhawk *Accipiter nisus*, northern goshawk *Accipiter gentilis*, kestrel *Falco tinnunculus*, black-headed gull *Chroicocephalus ridibundus*, common gull *Larus canus*, herring gull *Larus argenrtatus*, Eurasian magpie *Pica pica*, rook *Corvus frugilegus*, hooded crow *Corvus cornix* and raven. The observed mammalian predators were red fox and stoat *Mustela erminea*.

### Monitoring of lapwing nests

The approximate locations of nests were assessed by noting the position of incubating birds from distant vantage points. Subsequently, nests were located on foot. The location of each nest was marked in the field by a wooden stick placed ca. 10 m from the nest. In total, 61 nests were found and monitored.

When a nest was found during the laying period, its age was determined from the number of eggs laid, based on the assumption that a female lays a single egg each day. If the clutch was already completed, laying dates were back-calculated using egg floatation (van Paassen et al. 1984).

Nests were monitored every few days (median interval = 3 days; range 1–9) until hatching or nest failure. Hatching was determined by the presence of small eggshell fragments in the empty nest (Green et al. 1987). When a nest was found depredated (empty nest before the estimated hatching date and no eggshell fragments), it was assumed that the predation event occurred halfway between the last date the nest was observed active and the subsequent nest check.

### Measuring anti-predator behaviour

**Fig. 1:**
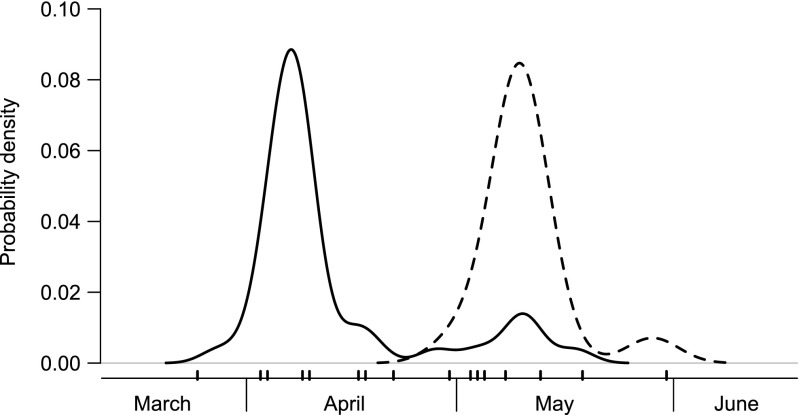
Seasonal timing within the studied lapwing population. *Lines* represent the kernel probability densities for first egg laying (*solid line*) and hatching (*dashed line*). Ticks on the horizontal axis denote the dates of the field visits

We considered the observed behaviour as mobbing when lapwings had undertaken a direct physical attack on a potentially dangerous predator. Observations were carried out from vantage points during 16 whole-day visits made between 25nd of March and 31st of May (Fig. 1). Only predators flying over the centre of the colony were considered as a potential threat. We measured the mobbing response by quantifying the maximum number of birds engaged in each mobbing event. In total, during 130 h of observations, data on 325 predator encounters were recorded.

### Data analysis

The daily survival rate of nests was calculated using the Mayfield method (Mayfield 1975). Overall nest survival was calculated for the median exposure time of 28 days found for our population.

We used generalized additive models (GAMs) to test the influence of timing (number of days after the 1st of March) and predator species on mobbing response. The GAM (Hastie and Tibshirani 1990) is an extension of the generalized linear model and allows for flexible, nonlinear and nonparametric functions to be included as predictors. The mobbing response was expressed as the proportion of the total number of adult lapwings present on the study area during each mobbing event that engaged in egg or chick defence. The total number of birds was estimated as twice the number of active nests on any given day. We considered all nests that successfully hatched as active because loses during hatching were exclusively partial at our study site, and following dispersal from the nest, it is unlikely that a predator would be able to locate all chicks from a brood.

We considered the three harrier species (marsh harrier, Montagu’s harrier and hen harrier), common buzzard, black-headed gull, rook, hooded crow and raven in the analysis. The other potential predators observed in the study area were excluded from the analysis due to small sample sizes. Observations of harriers were pooled into a single ‘harriers’ group for analysis, due to the relative similarity in hunting behaviour and small sample sizes.

We tested two candidate models; both included a categorical variable encoding the predator species/group and a smooth time term as explanatory variables. The first model, however, assumed additive relationships only. The second model included an interaction term and thus allowed for independent fits of the trend line for different predator species.

Models were fitted using a logit-link function and a binomial distribution for proportions. We used an information-theoretic approach to model selection and multi-model inference (Burnham and Anderson 2002). Two candidate models were compared using the Akaike Information Criterion, i.e. an estimate of the expected Kullback-Leibler information lost (Akaike 1974). The model with the lower AIC value was considered better, given the data, and the value of ΔAIC ≤ 2 was assumed as a threshold indicating models with substantial support (Burnham and Anderson 2002). Statistical analysis was performed in R version 3.2 (R Development Core Team 2015), using the mgcv 1.8 package (Wood 2006).

## Results

### Seasonal timing

Lapwings bred highly synchronously within the studied population (Fig. 1): The mode of laying dates fell on the 7th of April (interquartile range: 6th–11th of April), and the mode of hatching dates fell on the 10th of May (interquartile range: 9th–11th of May).

### Nest success

The proportion of successful nests was 52.9 % (95 % CI 39.4–66.2). The daily survival rate was 96.4 % (94.9–97.7) and overall nest survival was 36.2 % (22.8–51.6). For 21 nests (out of 61 monitored, 34.4 %), it was possible to infer the nest failure causes based on examination of tracks and nest remains. The main causes of nest failures were predation (57.1 %), nest abandonment due to frost (38.1 %) and trampling by cattle (4.8 %).

### Mobbing response

**Fig. 2:**
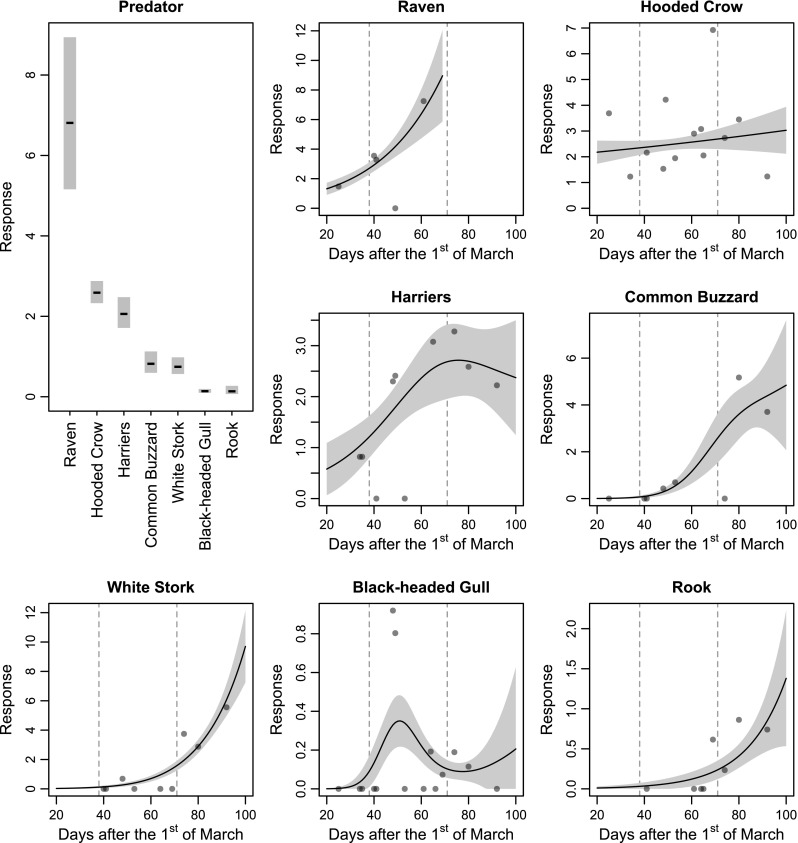
Generalized additive model results representing the effect of predators and smoothing curves for time on the mobbing response of lapwings against particular predator species. Time effects were modelled by separate fits for each predator. *Shaded regions* represent standard errors. *Vertical*, *dashed lines* are the median dates for egg laying and hatching. Response is expressed as % of mobbing lapwings to the estimated no. of all adult lapwings present within the study area

The mean response evoked by particular predators differed (Table 1, Fig. 2). The most highly mobbed species was raven, followed by hooded crow and harriers. Less mobbed were common buzzard, white stork, black-headed gull and rook.

**Table 1:** Parameters of the generalized additive model examining the intensity of lapwing mobbing behaviour (expressed as % of mobbing lapwings of the estimated no. of all lapwings present within the study area) in relation to predator species/group and date

Predator	Parametric terms (predators)			Smooth terms (temporal pattern)		
	Estimate	95 % LCI	95 % UCI	edf	Chi square	*P*
Raven	6.81	3.93	11.52	1.00	10.13	0.0015
Hooded crow	2.59	2.10	3.18	1.00	0.48	0.4889
Harriers	2.06	1.43	2.95	1.61	3.53	0.2089
Common buzzard	0.82	0.43	1.53	1.87	26.08	<0.0001
White stork	0.74	0.43	1.27	1.32	36.33	<0.0001
Black-headed gull	0.14	0.07	0.27	2.71	5.39	0.1682
Rook	0.13	0.03	0.53	1.00	4.38	0.0363

Predators are organized according to the column ‘estimate’, which is the mean response (% of birds engaged in mobbing behaviour). Estimated degrees of freedom, ‘edf’, reflect the smoothness of the fitted curve (one represents a straight line). The proportion of deviance explained by the model was 49.3 %

### Temporal patterns of defence

The model with the interaction term received the highest rank (AIC = 724.3). The model with additive effects received considerably less empirical support (AIC = 777.8, ΔAIC = 53.5, AIC weight < 0.001). Thus, the final inference was based only on the results of the interaction model.

Our results suggest that patterns of temporal variation in the mobbing response of lapwings were predator-dependent (Fig. 2). The mobbing response against raven, common buzzard, white stork and rook increased significantly throughout the breeding season (Table 1, Fig. 2). In the case of hooded crow, harriers and black-headed gull anti-predator response of lapwings did not exhibit any clear patterns (Table 1, Fig. 2).

## Discussion

Our results suggest that lapwings exhibit predator-dependent patterns of temporal variation in their mobbing response within the breeding season. Raven, hooded crow and harriers evoked the most intense overall response, while common buzzard, white stork and rook were markedly less attacked. This suggests that the mobbing behaviour of lapwings constitutes a flexible anti-predator strategy and birds match their response to the level of risk associated with encountering a predator.

Rook evoked scant reaction, which may suggest that lapwings perceive it as a harmless intruder. To our knowledge, none of the studies identified Rook as a predator of wader eggs or chicks. Rook is an omnivorous corvid species, but not a habitual egg predator, although it may occasionally plunder bird nests (Holyoak 1968). Some studies reported birds’ eggshells in Rooks diet (Orłowski et al. 2009, 2013), but the proportion of such item was low and Gromadzka (1980) identified these remains as poultry and in several cases duck eggshells, collected during the autumn-winter months. The mean response of lapwings against this species was markedly lower than against other potential predators with similar patterns of mobbing response. The significant increase of mobbing response against this species may result from higher aggression of lapwings post-hatching, with lapwings possibly more prone to attack even less hazardous species during this period.

The strongest defence displayed by lapwings was against raven, suggesting that it is perceived as the most hazardous predator, and the pattern of defence was consistent with the predictions of the reproductive value of offspring hypothesis. Ravens are capable of preying on fully grown Kittiwakes *Rissa tridactyla* (Klicka and Winker 1991) and Feral Pigeons *Columba livia* (Hendricks and Schlang 1998), and may threaten both young and adult lapwings. Furthermore, when a particular species is abundant, raven may specialise upon it and cause the majority of brood losses (Ratcliffe 1997; Colwell et al. 2012).

Hooded Crow is a common predator of wader eggs and sometimes chicks (Green et al. 1990), it is however rather unlikely to depredate older lapwing chicks. Thus, the hazard posed by Crows likely does not strongly increase throughout the breeding season. Lapwings may also expel Hooded Crows more effectively in collective defence, as this species is harmless to adults. Harriers constituted the third most mobbed predator type, and lapwings exhibited a nonlinear (though statistically insignificant) pattern of variation in mobbing response through the breeding season. Amar with co-workers (2008) found chicks of lapwings in harriers diet, and harriers are known to sometimes depredate waders eggs too (pers. observation).

Lapwings mobbed corvids and harriers also during the pre-laying period. Aerial acrobatics of males during courtship are similar to aerial dives on predators (Kis et al. 2000), and males display such mobbing behaviour to signal their parental capabilities (Grønstøl 1996; Parish and Coulson 1998; Liker and Székely 1999). Lighter (during the breeding period; Cramp and Simmons 1983) and more manoeuvrable males perform better in deterring predators and spend more time defending territory than females (Liker and Székely 1999). Thus, we hypothesize that mobbing bouts observed during the pre-laying period resulted from courtship behaviour.

Lapwings strongly increased their anti-predator response against Common Buzzard and White Stork after hatching, as chick age increased. Apparently, both species represent temporal predators, hazardous mainly to the chicks. These species are unlikely to depredate eggs but have been identified as important predators of wader chicks in some regions (Hönisch et al. 2008; Teunissen et al. 2008; Schekkerman et al. 2009). Similar patterns of brood defence have been found previously in black-tailed godwits *Limosa limosa*: birds attacked approaching common kestrels more intensively during rearing than during incubation (Green et al. 1990). Such a response supports the predictions of the reproductive value of offspring hypothesis.

Threat-sensitive defence likely allows lapwings to assess risk accurately and react appropriately. Individuals may balance their time and energy between predator avoidance and other activities, and avoid or minimize time and energy expenditure during less risky situations (Lima and Dill 1990; Sordahl 1990; Caro 2005; Ferrari et al. 2008). Such a flexible strategy possibly evolved as a response to living in a multi-predator environment, where different predators, displaying various hunting strategies, pose different hazards. Risk assessments by individuals may also be site-specific. Individuals may adjust the threat-sensitive defence to the structure of local predator communities (i.e. densities of particular species) and local predator pressure, as various predators may affect reproductive success of prey populations strongly at some sites or not at all at others (Krams et al. 2010; Sandoval and Wilson 2012). To date, the majority of studies concerning the ability to distinguish between predator types (species or guilds) and assess the associated risk to the chicks and adults (see Montgomerie and Weatherhead 1988; Caro 2005; Lima 2009 for review) have focused on behavioural responses during predator encounters in altricial species.

So far, the threat-sensitive predator avoidance hypothesis has been mostly considered independently of the reproductive value of offspring and harm to offspring hypotheses (e.g., Brown et al. 2006; Rhoades and Blumstein 2007; Mathot et al. 2009; Crawford et al. 2012), and only few studies have considered these hypotheses simultaneously (Ghalambor and Martin 2001; Schneider and Griesser 2015). Our results also suggest that these hypotheses are not mutually exclusive. If parents’ behaviour is risk-sensitive, then various patterns of temporal changes in mobbing response may emerge. Thus, a single prey species may exhibit behaviour consistent with different hypotheses derived from parental investment theory. In the case of most threatening predators, the anti-predator response may even be constant throughout the breeding period. We recommend that various predator species should be considered in studies exploring the variation in the anti-predator response and mobbing intensity to avoid biased results.

The observed proportion of birds that mobbed predators may increase throughout the breeding season due to a decreasing number of birds available at a nesting site. If mobbing is effective, we can predict that breeders that do not tend to mob would have their nest depredated as the breeding season progresses and therefore would not be considered to be present at the nesting site. However, it was not a case at our study site (see Supplementary Material). The differences in response of lapwings may also result from different encounter rates of each species. Due to the high energy costs of anti-predator responses, it is inefficient for birds to attack one abundant species each time it encounters it. For example, lapwings scarcely mobbed the black-headed gull, a species observed multiple times during each visit, which may explain the lack of a temporal pattern of defence.

In conclusion, we found that lapwings distinguish between different predator species and adjust mobbing response to reflect the threat posed by a given predator at different stages of the breeding cycle. This threat-sensitive predator avoidance likely allows lapwings to optimize the trade-off between offspring defence and other activities.

## Electronic supplementary material

Below is the link to the electronic supplementary material.

ESM 1 (DOC 51 kb)
