# Supplementary material for: Threat-sensitive anti-predator defence in precocial wader, the northern lapwing Vanellus vanellus
Source: Acta Ethol. 2016 May 19;19(3):163–71. doi: 10.1007/s10211-016-0236-1 (PMC5039224; doi:10.1007/s10211-016-0236-1)
Supplement: Supplementary file 1 — (DOC 51 kb) [file 10211_2016_236_MOESM1_ESM.doc]

**Relationship between the number of mobbing Lapwings and time of the season**

If mobbing is effective, we can predict that as the breeding season progresses, breeding Lapwing that do not tend to mob would have their nest depredated and therefore would not be considered to be present at the nesting site. As a result, the higher proportion of Lapwings engaging in mobbing behaviour could be due to a decrease in the number of non-mobbing Lapwing and not due to an increased mobbing response. In other words: the observed proportion may increase not due to increasing number of “successes” (no. of birds that mob, when the generating process is treated as a sample drawn from the binomial distribution), but due to decreasing number of draws (no. birds available).

We checked this presumption by fitting a GAM model using the number of mobbing Lapwings as a response, assuming the negative binomial distribution for counts and log-link function (Table S1). The temporal pattern for counts is significant and strictly linearly increasing (Figure S1), which contradicts the hypothesis that the increasing proportion of mobbing birds is due to decreasing no. of birds available. This result suggests that Lapwings tend to mob in greater numbers as the season progresses, independent of the number of nests that are lost due to predation.

**Table S1.** Results of GAM examining the effect of Julian date on the number of mobbing Lapwings.

| Parametric coefficients: | | | | |
| --- | --- | --- | --- | --- |
|  | Estimate | Std. Error | z value | p value |
| Intercept | -0.3637 | 0.3062 | -1.188 | 0.235 |
| Harriers | 0.7256 | 0.4096 | 1.772 | 0.076 |
| White Stork | 0.3554 | 0.3771 | 0.942 | 0.346 |
| Raven | 1.2753 | 0.3392 | 3.760 | < 0.001 |
| Hooded Crow | 1.8642 | 0.4288 | 4.348 | < 0.001 |
| Rook | -1.3789 | 0.5040 | -2.736 | 0.006 |
| Black-headed Gull | -1.8574 | 0.4171 | -4.453 | < 0.001 |
| Smooth terms: | | | | |
|  | Edf | Ref.df | Chi.sq | p-value |
| s(julian) | 1.146 | 1.276 | 8.234 | 0.005 |
|  |  |  |  |  |
|  | | | | |


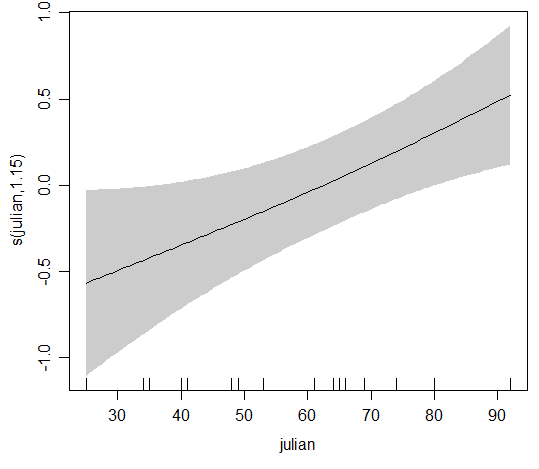


**Figure S1.**  GAM results showing the effect of Julian date on the number of mobbing Lapwings. Shaded regions represent standard errors.
